# The efficacy and tolerability of sports drink versus water in bowel preparations: a randomised controlled study

**DOI:** 10.1186/s13063-022-06658-2

**Published:** 2022-08-26

**Authors:** Zhixin Zhang, Hui Gao, Xin Yuan, Cenqin Liu, Zhenfei Bao, Siyi Yu, Haofen Xie, Weihong Wang, Jiarong Xie, Lei Xu

**Affiliations:** 1 Department of Gastroenterology, Ningbo First Hospital, Ningbo, 315010 China; 2 College of Medicine, Ningbo University, Ningbo, 315211 China; 3 College of Medicine, Zhejiang University, Hangzhou, 310058 China

**Keywords:** Bowel preparation, Colonoscopy, Mizone, Polyethylene glycol, Tolerability

## Abstract

**Background:**

An optimal bowel preparation can result in an improved colonoscopy. This study was to compare the effectiveness and safety of the use of a sports drink (Mizone) plus polyethylene glycol (PEG) solution with a water plus PEG solution in bowel preparations.

**Methods:**

This was a randomised controlled study. All of the included patients were randomly divided into the following two groups: the PEG + Mizone group and the PEG + water group. The palatability of the solution was measured through the use of questionnaires. Additionally, bowel cleanliness was evaluated according to the Ottawa Bowel Preparation Scale (OBPS, 0–14, with higher values indicating worse cleanliness), as well as with the aid of colonoscopy videos.

**Results:**

A total of 270 patients were enrolled. The rate of adequate bowel preparation was 74.8% in the PEG + Mizone group and 68.9% in the PEG + water group, with a risk difference of 5.9% (95% CI: − 4.8–16.6%), which indicated noninferiority (noninferiority margin: − 9.5% <  − 4.8%). However, patients rated the palatability (65.9% vs 44.4%, *P* < 0.001) and willingness to recommend or repeat (88.9% vs 75.6%, *P* = 0.004) the administration of the PEG + Mizone preparation as being better than those of the PEG + water preparation. The rates of adverse events during the bowel preparations were not significantly different between the two groups, except for bloating (PEG + Mizone vs PEG + water, 4.4% vs 13.3%, *P* = 0.010).

**Conclusion:**

The concomitant use of PEG + Mizone was a well tolerated and effective bowel preparation, compared with the PEG + water treatment.

**Trial registration:**

ClinicalTrials.gov NCT04247386. Registered on 30 Jan 2020.

**Supplementary Information:**

The online version contains supplementary material available at 10.1186/s13063-022-06658-2.

## Background

A colonoscopy plays an important role in reducing the incidence and mortality of colorectal cancer [1], and adequate bowel preparations represent one of the most crucial factors for a complete colonoscopy [2]. A good bowel preparation will lead to shorter caecal intubation times, better visualisations of the mucosa and better polyp detections [3, 4]. Polyethylene glycol (PEG) is one of the most common laxatives that are used in bowel preparations for colonoscopies because of its safety [5]. However, patients may demonstrate nausea, vomiting, abdominal pain and distension due to the large amount of fluid consumption and bad taste; thus, the patients cannot even complete the bowel preparation. Furthermore, an unpleasant experience may lead to the reluctance of the patients to receive a repeated colonoscopy.

Many relevant studies have demonstrated that PEG powder mixed with a tasteful solution (such as green tea and Gatorade [6, 7]) may be a modified method for the performance of a bowel preparation, and the use of seasoning can improve palatability without reducing bowel cleanliness. Furthermore, patients also tend to possess higher levels of tolerance [8]. However, it is unknown as to whether other drinks can exert similar effects. Therefore, we hypothesised that Mizone, which is a sports drink with a high degree of popularity and a first brand power index in China (and which also contains various vitamins), may be good for health as a flavouring agent for imparting extra flavour to PEG. We performed a prospective, randomised controlled trial to explore the potential effects of Mizone on bowel preparations through the combined application of PEG.

## Materials and methods

### Study design

This was a prospective, single-centre, operator- and assessor-blinded randomised clinical trial. The trial complied with the Declaration of Helsinki, written informed consent was obtained from the patients and the study protocol was approved by the Ningbo First Hospital Institutional Ethics Committee (2019-R065). The study was registered at Clinical Trials.gov (NCT 04,247,386) on 30/1/2020.

### Patients

Outpatients aged 18–75 years old, who required colonoscopies and were willing to participate in this study between December 2019 and August 2020 were consecutively enrolled. The following exclusion criteria were used: (1) patients with constipation; (2) patients with severe liver, kidney and heart dysfunction; (3) patients with a history of poor intestinal preparation; (4) pregnant/lactating women; (5) patients who were allergic or intolerant to any type of research drug; (6) patients with severe gastrointestinal diseases, such as intestinal obstructions or perforations, toxic colitis and toxic megacolon; (7) patients with a history of inflammatory bowel disease; (8) patients with significant electrolyte anomalies, including abnormalities in phosphorus, sodium, potassium, calcium, chloride and magnesium levels; (9) patients with a history of colorectal resections; and (10) patients with diabetes. After receiving a full explanation of the study, all of the patients were provided with a detailed oral education and written informed consent before the recruitment.

### Endoscopic procedure

All of the patients were instructed to adhere to a 1-day low-residue diet before the colonoscopy [9–11]. Patients who were allocated into the PEG + Mizone group (experimental group) received 180 g PEG with 1.2 L clear water plus 1.8 L Mizone liquids, whereas the patients in the PEG + water group (control group) received 180 g PEG with 3 L clear water for the bowel preparations. They were instructed to ingest one-third of the solution (60 g PEG + 1 L Mizone liquids plus clear water/clear water) between 9 and 10 pm on the day before the colonoscopy, after which they would ingest the remaining two-thirds of the solution (120 g PEG + 2 L Mizone liquids plus clear water/clear water) at 4–6 h before the colonoscopy. Intestinal cleanliness was evaluated according to the video by a specific assessor of outcomes, other information (such as patient age, height, weight, advent events and willingness) was collected by another assessor during the colonoscope, and operator was only responsible for colonoscopy. Partial blinding of the participants was applied (blinding of two assessors of outcomes, blinding of the operator, but patients in this trial were nonblinded).

All of the procedures were performed with the use of the same model of high-definition video colonoscope (CF- H290 or CF- HQ290 video colonoscope, Olympus Co, Tokyo, Japan) by a single experienced endoscopist, and all of the patients underwent the colonoscopies with air.

### Study medications

PEG powder (Hengkang Zhengqing, Jiangxi Hengkang Pharmaceutical Co. Ltd., China) was packed into a box that included 3 bags of reagents A, B and C. Each bag of reagent A consisted of 1.68 g of sodium bicarbonate and 0.74 g of potassium chloride. Each bag of reagent B contained 5.68 g of sodium sulphate and 1.46 g of sodium chloride. Each bag of reagent C contained 60 g of PEG 4000. A total of three boxes of PEG powder and 3 L clear water were used to produce 3 L PEG solution, whereas a total of three boxes of PEG powder with 1.2 L clear water and 1.8 L Mizone (Danone China Food & Beverage) were used to produce 3 L PEG-Mizone solution.

### Outcomes

The primary endpoint in this study was the degree of colonic cleanliness in the PEG + water group and the PEG + Mizone group. Colonic cleanliness was measured according to the Ottawa Bowel Preparation Scale (OBPS) [12]. The OBPS consists of two parts, colon segment (right: cecum, ascending; mid: transverse, descending; and rectosigmoid) and fluid quantity. Colon segment was scored on a four-point scale (0–4), fluid quantity was scored on a three-point scale (0–2). Two parts scores were summed to yield a total score (range, 0–14, higher is worse). With the use of the OBPS, bowel preparation was considered to be “adequate” when the total score was less than 7. Secondary endpoints included the patients’ tolerabilities of the bowel preparations (including palatability, repeat willingness to undergo another preparation and the rates of completeness of the administrated liquids) and the rate of adverse events (including electrolyte disorders that required clinical treatment, nausea, vomiting, abdominal pain and bloating). Each patient completed a form regarding their palatability for the bowel preparation solution. The form was scored by using a modified 5-point Likert scale for ease of use (scores 5–1: very good, good, neutral, bad or very bad, respectively), and palatability was considered to be “good” when the score was greater than 3. All of the patients received follow-ups via telephone within 3 days to assess the frequency of adverse postoperative events. Only the electrolyte disorders that required clinical treatments were regarded as being adverse events.

### Sample size

The noninferiority test was used to calculate the sample size. The rate of adequate bowel preparation (Boston bowel preparation scale ≥ 6) of 92.5% has been reported in an article in 2019 [13]. By comparison, the rate of adequate bowel preparation (Ottawa bowel preparation scale < 7) of 83% has been reported in another article in 2011 [14]. The absolute risk difference between the two researches was 9.5%, we hypothesised that PEG was 92.5% efficacious for both groups in achieving an adequate score, the non-inferiority margins for PEG + Mizone compared with PEG + water were defined at 9.5% in absolute risk difference (RD), in order to ensure that the “adquete rate” for the PEG + Mizone group would exceed 83%. Based on a 2-sided significance level of 0.025 and a power of 80%, the estimated sample size was determined to be 121 patients in each arm. Additionally, we assumed that approximately 10% of the patients would eventually be excluded from the analysis set; thus, a total sample size of 270 patients would be needed for the study.

### Randomisation and masking

A simple randomisation strategy was used, and the patients were randomly assigned at a 1:1 ratio to the two treatment groups by using Stata (version 13.0, Stata Corp LP, College Station, TX). Subsequently, the generated randomised sequence with a serial number was assigned to an opaque, sequentially numbered envelope by a staff member who was unaffiliated with the study. The allocation table was concealed from the operators. Intestinal scoring was performed by a proficient independent observer, according to the video.

### Statistical analysis

Statistical analyses were performed by using SPSS (version 21, SPSS, Chicago, IL, USA). All of the included patients were enrolled in the intention to treat (ITT) analysis. The primary endpoint was also analysed according to the per-protocol (PP) analysis principle. In regard to the efficacy of the bowel cleansing, if the lower bound of the 95% confidence interval (95% CI) of the RD was greater than − 9.5%, then the noninferiority of the PEG + Mizone group could be concluded. We analysed the categorical outcomes by using Fisher’s exact test and compared the continuous outcomes by using the Mann–Whitney *U* test. Results with *p*-values < 0.05 (two-sided) were considered to indicate significance.

## Results

### Patient characteristics

**Fig. 1:**
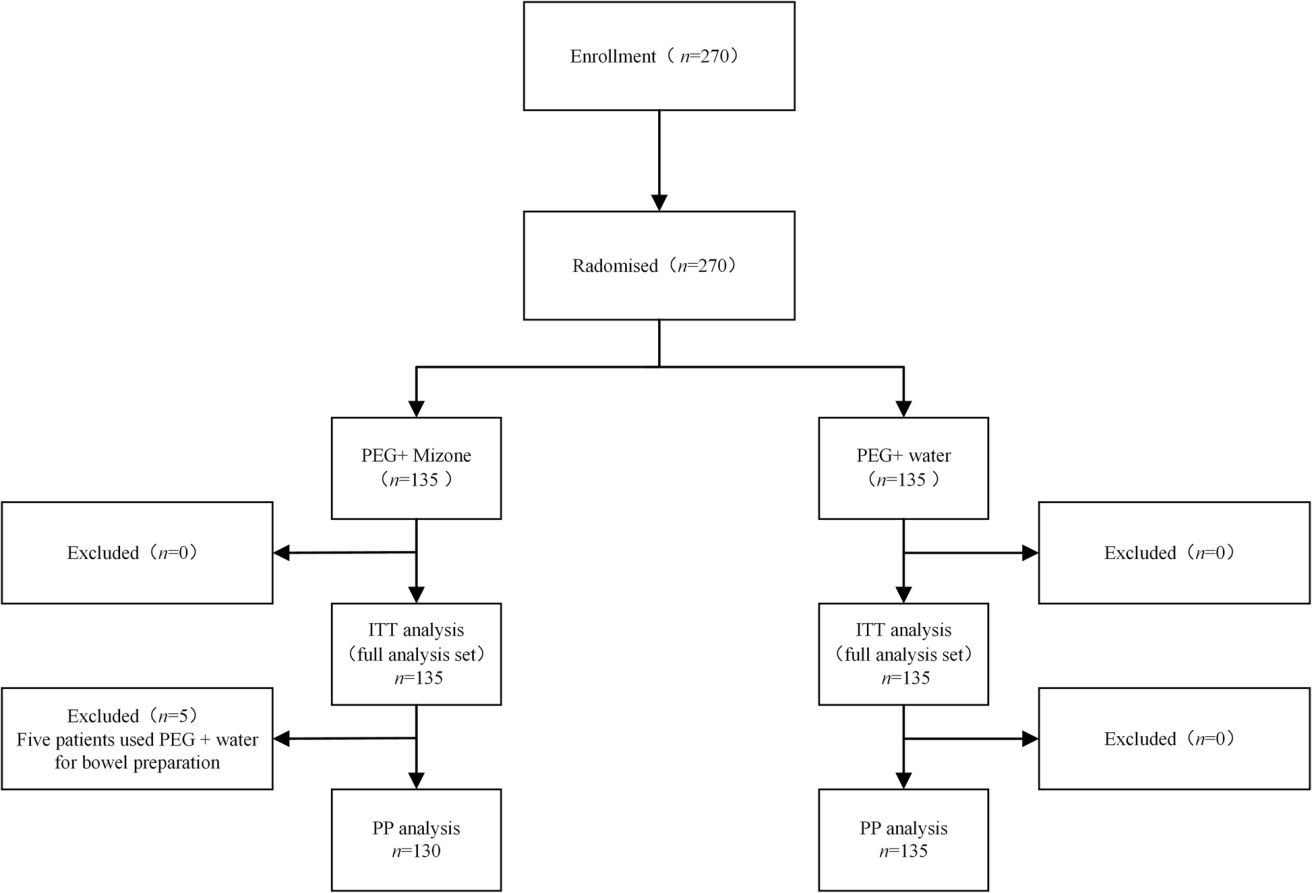
Study flowchart

From December 2019 to August 2020, we enrolled 270 patients and randomly assigned them to either the PEG + Mizone group (*n* = 135) or to the PEG + water group (*n* = 135). Thus, a final total of 270 patients were included in the ITT analysis for the primary endpoint. Five patients in the PEG + Mizone group and no patients in the PEG + water group were excluded because of protocol violations (not following the instructions for bowel preparations), resulting in 265 patients (130 patients in the PEG + Mizone group and 135 patients in the PEG + water group) being included in the PP analysis (Fig. 1). Table 1 demonstrates that no significant differences were observed between the two groups in regards to the demographic data, indications for colonoscopy and whether the patients were receiving a colonoscopy for the first time or not. Additionally, 38 experienced patients (80.9%) in the PEG + Mizone group demonstrated that this bowel preparation was better than the previous preparation (PEG + water).

**Table 1:** Demographics of patients in the two groups

Variable	PEG + Mizone (*n* = 135)	PEG + water (*n* = 135)	*P-*value
Age, mean (SD), years	54.7 ± 11.9	57.1 ± 11.7	0.095
Gender, *n* (%)			
Male	59 (43.7)	70 (51.9)	0.223
Female	76 (56.3)	65 (48.1)	
Height, mean (SD), cm	165.2 ± 7.8	164.4 ± 7.4	0.369
Weight, mean (SD), kg	61.8 ± 11.2	62.2 ± 10.7	0.751
BMI, mean (SD), kg/m2	22.5 ± 2.9	22.9 ± 2.7	0.256
Current drinking, *n* (%)			
Yes	22 (16.3)	30 (22.2)	0.217
No	113 (83.7)	105 (77.8)	
Current smoking, *n* (%)			
Yes	26 (19.3)	25 (18.5)	> 0.999
No	109 (80.7)	110 (81.5)	
Indications for colonoscopy, *n* (%)			0.964
Screening	43 (31.9)	39 (28.9)	
Polyp followup	37 (27.4)	40 (29.6)	
Family history of CRC	7 (5.2)	6 (4.4)	
Abdominal pain/discomfort	23 (17.0)	26 (19.3)	
Bloody stool	25 (18.5)	24 (17.8)	
Non-first-time colonoscopy, *n* (%)	47 (34.8)	55 (40.7)	0.315

*BMI* body mass index, *PEG* Polyethylene glycol, *CRC* colorectal cancer, *SD* Standard deviation

### Primary endpoint

**Fig. 2:**
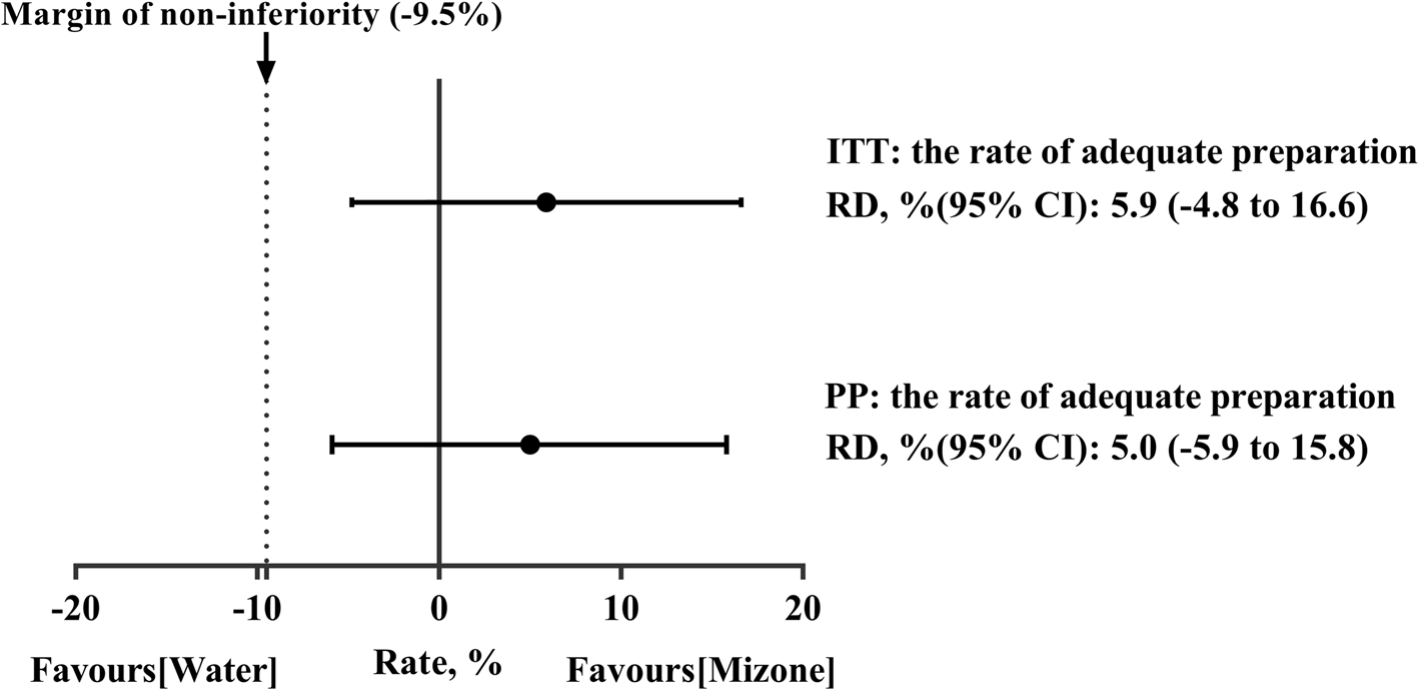
Non-inferiority of the PEG + Mizone regimen relative to the PEG + water regimen. ITT, intention-to-treat; PP, per-protocol; RD, risk difference. Adequate preparation: Ottawa Bowel Preparation Scale < 7

The ITT analysis demonstrated that the rate of adequate bowel preparations (OBPS < 7) was 74.8% (101/135 patients) in the PEG + Mizone group and 68.9% (93/135 patients) in the PEG + water group, with a RD of 5.9% (95% CI: − 4.8–16.6%) (noninferiority margin: − 9.5% <  − 4.8%). In the PP analysis, the results were equivalent between the PEG + Mizone group (73.8%, 96/130 patients) and the PEG + water group (68.9%, 93/135 patients), with a RD of 5.0% (95% CI: − 5.9–15.8%) (noninferiority margin: − 9.5% <  − 5.9%) (Fig. 2). The means of the total and each intestinal OBPS scores exhibited no significant differences between the PEG + Mizone group and the PEG + water group in the ITT and PP analyses (ITT, total 5.3 ± 1.7/5.6 ± 1.6, *P* = 0.167. PP, total 5.4 ± 1.7/5.6 ± 1.6, *P* = 0.249; Figure S1). In general, the results indicated non-inferiority between the groups when regarding the rate of adequate bowel preparations in the ITT and PP analyses.

### Secondary endpoints

**Fig. 3:**
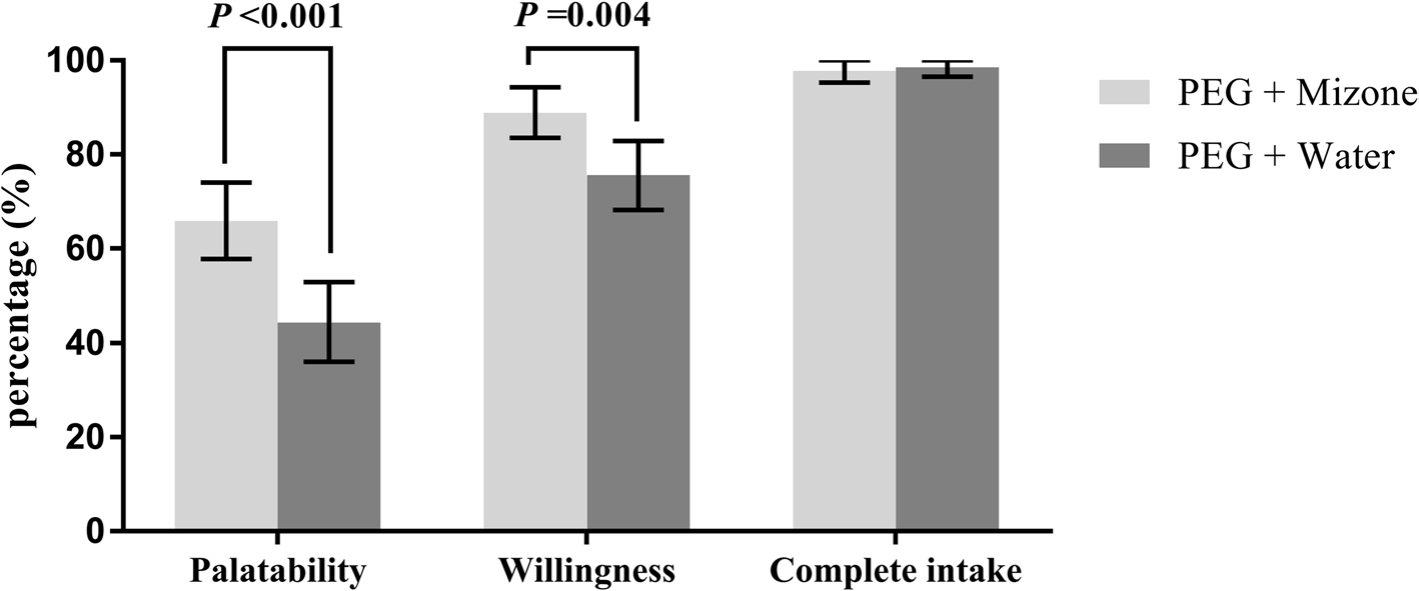
The incidence of secondary endpoints. Error bars represent 95% confidence intervals

**Fig. 4:**
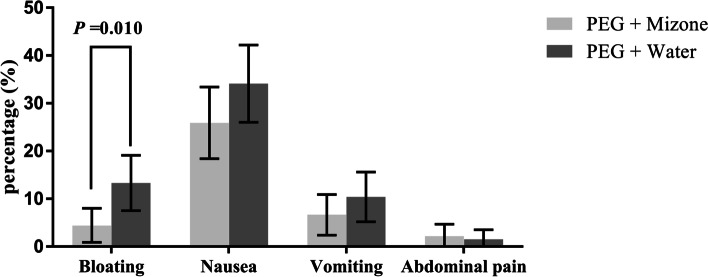
The incidence of advent events. Error bars represent 95% confidence intervals

The rate of “good” palatability scores in the PEG + Mizone group was significantly better than the rate in the PEG + water group (65.9% vs 44.4%, *P* < 0.001, Fig. 3). The rate of willingness to recommend or repeat the procedure was higher in the PEG + Mizone group than in the PEG + water group (88.9% vs 75.6%, *P* = 0.004, Fig. 3). The incidence of bloating in the PEG + Mizone group was significantly better than in the PEG + water group (4.4% vs 13.3%, *P* = 0.010, Fig. 4), whereas there were no significant differences in the incidences of the other three adverse events (including nausea, vomiting and abdominal pain) (Fig. 4). In this study, the proportions of subjects who completely ingested the laxative were not significantly different between the two groups (97.8% vs 98.5%, *P* > 0.999, Fig. 3). Although ten patients were lost to follow-up (six patients in the PEG + Mizone group and four patients in the PEG + water group), the remaining patients had no serious adverse events that needed clinical treatment.

## Discussion

Our randomised controlled trial demonstrated that efficacy and safety were not inferior for the PEG + Mizone treatment compared with the PEG + water treatment. Unsurprisingly, for palatability, the PEG + Mizone treatment was better than the PEG + water treatment, with a higher degree of willingness to repeat the procedure and the vast majority of experienced patients considering the PEG + Mizone preparation to be better than the previous preparation. Therefore, we determined that PEG + Mizone can be used as one of the standard methods for bowel preparations, especially in the Chinese or East Asian populations.

When considering bowel cleanliness, there was no difference in the total OBPS scores. This result is likely due to the high compliance rates in both groups. In addition, we adopted the split-dose regimen for bowel preparations, and the time interval between bowel preparation education and the colonoscopy was short [15, 16]. Similar to our results, previous studies have demonstrated that the addition of adjuvants may not affect the efficacy of the PEG solution [17–19]. Although no statistically significant differences were evidenced between the two groups, PEG + Mizone demonstrated an obvious trend (73.8% vs 68.9%).

In terms of the selection of the preparation regimens, patients preferred preparations that were lower in volume, more palatable and easier to complete. In our study, the mean palatability score was noticeably better in the PEG + Mizone group than in the PEG + water group, and the former group was more willing to choose the same regimen again. Only five patients in the PEG + Mizone group described the preparation as being too sweet to completely ingest; thus, they switched to the PEG + water treatment. Forty-seven patients in the PEG + Mizone group had previously received colonoscopies, and 38 (80.9%) patients considered this preparation regimen to be better than the previous regimen (PEG + water). Therefore, we believe that the PEG + Mizone group achieved higher acceptability among patients because of the better taste of this preparation. We assume that the use of Mizone is not cumbersome and does not increase the volume for bowel preparations.

There was no difference in the incidences of adverse events in both groups; instead, PEG + Mizone exhibited better results in terms of bloating. Prior research showed that the addition of sports drinks did not seem to cause a noticeable change in the levels of electrolytes [20]. Although no serological test was conducted in our research, there were no additional adverse events in the three days following the study, based on our follow-up results. Therefore, we deduce that diluted Mizone was observed to not cause unanticipated electrolyte disturbances. Based on the previously mentioned facts, the PEG + Mizone regimen appeared to be safe, and however, more prospective studies on the evidence of electrolyte changes are still needed.

Based on a previous study [21], one strength of our study is that we videotaped the colonoscopies of all of the patients and assessed the OBPS scores with one blinded researcher (with the scores based on the colonoscopy video) to ensure the reliability of the results. This avoided the risk of a breakdown of the blinding factor during the procedure that the researchers were grading (the operator and another assessor were not involved in the evaluation due to the fact that the patients may unintentionally reveal their preparation method), thus making our results more objective. We hope that our study may make a contribution to the improvement of bowel preparations.

Our study also had limitations. First, due to the limitations on research funds and time in the present study, we did not perform serological examinations. Although we did not determine electrolyte changes in patients and there could be certain risks in high-risk groups, no adverse events that required clinical interventions were observed. Second, this was a single-centre study with a limited number of patients, and all of the patients who were included in this study were Chinese. Therefore, it is possible that the results may not be generalizable. Third, a portion of the data was obtained by using questionnaires; thus, a recall bias may have occurred. However, we believe that this bias does not affect our conclusions because this problem existed in both groups.

## Conclusions

In conclusion, the combination of Mizone with PEG powder is a safe, well tolerated and acceptable preparation for patients to receive prior to a colonoscopy, and it did not reduce bowel cleanliness.

## Supplementary Information

**Additional file 1:** **Figure S1.** The OBPS score (mean) in the ITT and PP analysis.

## Data Availability

The datasets used and/or analysed during the current study are available from the corresponding author on reasonable request.

## Abbreviations

BMI: Body mass index
CI: Confidence interval
CRC: Colorectal cancer
ITT: Intention to treat
OBPS: Boston Bowel Preparation Scale
PEG: Polyethylene glycol
PP: Per-protocol
RD: Risk difference
SD: Standard deviation
