# Supplementary figures and images for: The efficacy and tolerability of sports drink versus water in bowel preparations: a randomised controlled study
Source: Trials. 2022 Aug 26;23:709. doi: 10.1186/s13063-022-06658-2 (PMC9419325; doi:10.1186/s13063-022-06658-2)

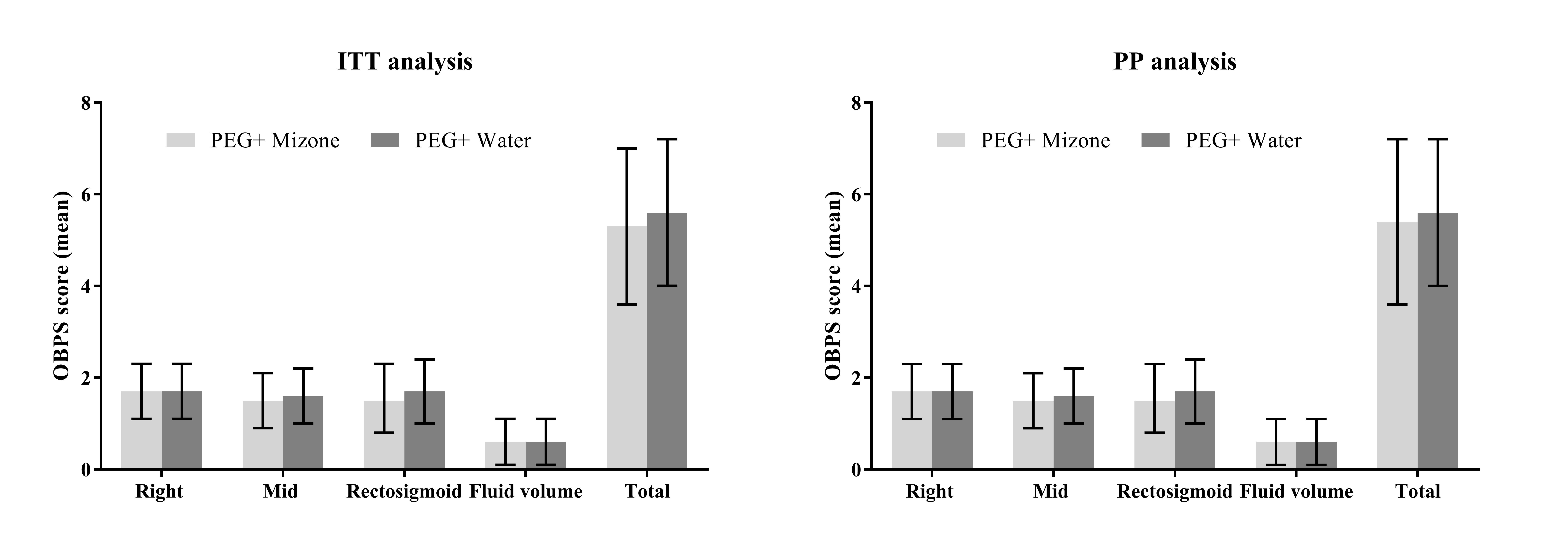

Supplement: Supplementary file 1 — Additional file 1: Figure S1. The OBPS score (mean) in the ITT and PP analysis. [file 13063_2022_6658_MOESM1_ESM.tif]
